# Jealousy in women with migraine: a cross-sectional case-control study

**DOI:** 10.1186/s10194-020-01114-5

**Published:** 2020-05-11

**Authors:** Daphne S. van Casteren, Florine A. C. van Willigenburg, Antoinette MaassenVanDenBrink, Gisela M. Terwindt

**Affiliations:** 1 Department of Internal Medicine, Erasmus University Medical Center, PO Box 2040, 3000 CA, Rotterdam, The Netherlands; 2 Department of Neurology, Leiden University Medical Center, PO Box 9600, 2300 RC, Leiden, The Netherlands

**Keywords:** Migraine, Jealousy, Estrogen

## Abstract

**Background:**

Estrogen influences susceptibility to migraine attacks and it has been suggested to affect jealousy in romantic relationships in women. Therefore, we hypothesized that migraine women may be more jealous.

**Methods:**

Jealousy levels and hormonal status were determined based on a cross-sectional, web-based, questionnaire study among female migraine patients and controls. A random sample of participants was selected from a validated migraine database. Participants with a serious and intimate monogamous relationship were included (*n* = 498) and divided into the following subgroups: menstrual migraine (*n* = 167), non-menstrual migraine (*n* = 103), postmenopausal migraine (*n* = 117), and premenopausal (*n* = 57) and postmenopausal (*n* = 54) controls. The primary outcome was the difference in mean jealousy levels between patients with menstrual migraine, non-menstrual migraine and premenopausal controls. Results were analyzed with a generalized linear model adjusting for age, relationship duration and hormonal status (including oral contraceptive use). Additionally, the difference in jealousy levels between postmenopausal migraine patients and controls was assessed. Previous research was replicated by evaluating the effect of combined oral contraceptives on jealousy.

**Results:**

Jealousy levels were higher in menstrual migraine patients compared to controls (mean difference ± SE: 3.87 ± 1.09, *p* = 0.001), and non-menstrual migraine patients compared to controls (4.98 ± 1.18, *p* < 0.001). No difference in jealousy was found between postmenopausal migraine patients and controls (− 0.32 ± 1.24, *p* = 0.798). Women using combined oral contraceptives were more jealous compared to non-users with a regular menstrual cycle (2.32 ± 1.03, *p* = 0.025).

**Conclusion:**

Young women with migraine are more jealous within a romantic partnership.

## Background

Sex hormones have a major influence on migraine, appearing from a three times higher migraine prevalence in premenopausal women compared to men, an increase in attack frequency during menopausal transition, and a postmenopausal decrease of symptoms [1–3]. Furthermore, the fluctuation of estrogen prior to menstruation is evidently linked to an increased susceptibility to an upcoming attack [4]. Two subtypes of migraine with menstruation-associated attacks exist: pure menstrual migraine (PMM) and menstrually-related migraine (MRM). In MRM, attacks occur additionally at other times of the cycle. For research purposes, PMM and MRM are often taken together and defined as menstrual migraine (MM) [5]. Although the exact pathophysiological underlying mechanism remains unclear, previous research has suggested that fluctuations in estrogen levels, possibly the rate of decrease in estrogen, may affect the susceptibility to migraine attacks in women and/or higher estrogen levels may be implicated in both sexes [2, 6–9].

Problems within a romantic relationship, such as jealousy, divorce, and bereavement after the suicide of a partner potentially have a large impact on quality of life [10, 11]. Knowledge on potential associations between relationship problems and disabling chronic diseases, such as migraine, may increase our understanding, reduce stigma, and improve disease outcomes. Relationship jealousy can be defined as thoughts, emotions, or behaviors that occur as a result of the perceived threat of losing a partner to an actual or imagined rival [12]. In the fertile phase, when estrogen levels are high, women tend to report higher jealousy levels compared to other times of the menstrual cycle [13]. Furthermore, jealousy seems to be affected by the use of combined oral contraceptives. Especially using formulations with higher doses of ethinyl estradiol are associated with significantly higher jealousy scores [13–15]. These findings indicate that estrogen plays a role in jealousy within a romantic relationship, but the exact underlying mechanism is unknown.

That biological factors may affect mental health has been illustrated by previous research concluding that alterations in prolactin and thyroid hormone levels are associated with suicide attempts in psychiatric patients [16].

We hypothesized that women with migraine, especially those fulfilling the criteria of MM, would have higher jealousy levels compared to women with non-menstrual migraine (non-MM) and premenopausal controls due to a corresponding provoking effect of estrogen in migraine and jealousy. Secondarily, we hypothesized that postmenopausal migraineurs and controls report low and similar jealousy levels due to stabilization of sex hormones [3, 17]. Lastly, we investigated the effect of using combined oral contraceptives on jealousy, aiming to replicate previous results on this topic.

## Methods

### Study design

This study is a cross-sectional, web-based, questionnaire study among female migraine patients and healthy controls, performed in November and December 2018.

### Participants

The Leiden University Medical Center MIgraine Neuro Analysis (LUMINA) cohort was used to select women who met the ICHD-3 criteria for migraine and healthy controls [5]. An elaborate description of LUMINA participants and procedures is found in a previous publication and in Additional file 1 [18]. The study was approved by the medical ethics committee of Leiden University Medical Center. All subjects provided written informed consent prior to the study. A random selection of *n* = 1024 female migraine patients and controls was made from the LUMINA cohort for this present study.

As inclusion criterium participants were required to have a serious and intimate monogamous relationship, assuming that contributions are equally divided among the partners and both partners have a concern for the welfare of the other, and will therefore respond to each other’s needs [19]. Pregnant and breastfeeding women were excluded. Additionally, women with a permanent primary amenorrhea, and therefore lifelong absence of menses, were excluded. Participants received a web-based questionnaire consisting of questions concerning relationship duration, jealousy feelings and thoughts, menstrual cycle status and exogenous sex hormone use. Jealousy scores were determined using the validated Buunks Jealousy scale (Cronbach’s alpha = 0.843) [12]. This questionnaire consists of five statements for each of the three sub-types of jealousy, i.e. reactive jealousy (a negative response to the emotional or sexual involvement of the partner with someone else), preventive jealousy (efforts to prevent intimate contact of the partner with someone else) and anxious jealousy (obsessive anxiety and worrying about the possibility of infidelity of the partner). A 5-point Likert scale was used to rate how strongly the participants agreed with the statements. Premenopausal migraine patients were categorized as MM or non-MM according to the ICHD-3 criteria [5].

### Covariates

The covariates age, relationship duration and hormonal status were chosen a-priori based on previous studies. Relationship duration was categorized as shorter or longer than 1 year. Although the effect of relationship duration on jealousy levels is inconsistent in previous studies, this covariate was reasoned to be important, and therefore, was included in this study [20, 21]. Hormonal status was defined as the use of combined oral contraceptives (COC), use of other hormonal contraceptives or no use of hormonal contraceptives (i.e. naturally menstruating). Other hormonal contraceptives included desogestrel-only pills, levonorgestrel intrauterine devices, etonogestrel subcutaneous implants, medroxyprogesterone injections and an ethinylestradiol/​etonogestrel ring. The naturally menstruating group consisted of women with a regular menstrual cycle (i.e. duration of 21 to 35 days) or irregular menstrual cycle (i.e. shorter than 21 days, longer than 35 days or irregular). The use of hormonal contraceptives has been shown to increase jealousy levels and was an important covariate to include in our analyses [13, 14].

### Statistical analyses

One-way ANOVA or Chi-square tests were used to compare the characteristics between the different groups. For our primary analysis we performed a generalized linear model to assess the mean difference between the total self-reported jealousy scores of MM, non-MM and premenopausal controls. Age, relationship duration and hormonal status were included as covariates. In a secondary analysis, we compared the mean total jealousy levels of postmenopausal migraine patients and controls using a generalized linear model, adjusting for age and relationship duration. The same statistical model was used to compare mean jealousy levels between women using COC and women with a regular menstrual cycle, controlling for age, relationship duration and migraine status. Mean differences in sub-type jealousy levels were analyzed for the premenopausal groups as exploratory analyses. A *p*-value of < 0.05 was considered statistically significant.

## Results

### Participants

**Fig. 1:**
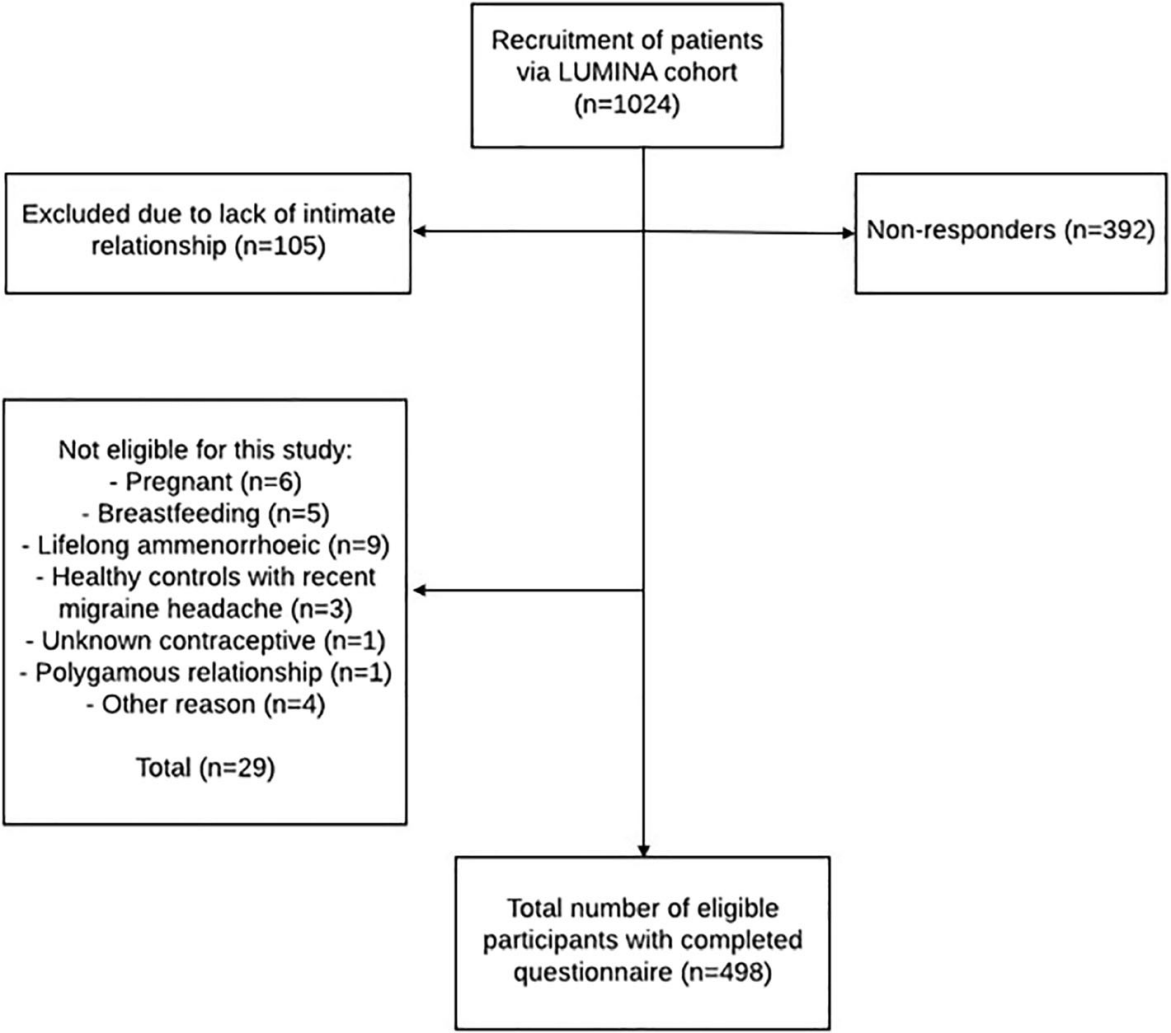
Flow diagram of recruitment of participants

A total of 1024 women were invited to participate in this study, of which 498 were eligible and completed the questionnaire (see Fig. 1).

The characteristics of the premenopausal and postmenopausal study populations are shown in Tables 1 and 2, respectively. The majority of premenopausal migraine patients was classified as MM (62%), of which 38% fulfilled the criteria of migraine with aura. In the non-MM group, 60% of patients had migraine with aura. The number of migraine days per month was higher in women with MM compared to women with non-MM, with at least one migraine day per week in 38% of the MM group compared to 20% in the non-MM group. In the postmenopausal migraine group, 39% experienced at least one migraine day per week.

**Table 1:** Characteristics of the premenopausal study population

	Control (*n* = 57)	non-MM (*n* = 103)	MM (*n* = 167)	*p*-value
Age, y, mean (SD)	37.2 (9.7)	37.5 (10.3)	38.8 (9.3)	0.401
Relationship duration > 1 year, n (%)	50 (87.7)	89 (86.4)	160 (95.8)	0.015
BMI, mean (SD)	23.1 (3.7)	23.5 (4.1)	23.7 (4.0)	0.594
Menstrual cycle, n (%)	29 (50.9)	31 (30.1)	102 (61.1)	< 0.001
Regular menstrual cycle	24 (42.1)	24 (23.3)	68 (40.7)	
Irregular menstrual cycle	5 (8.8)	7 (6.8)	34 (20.4)	
COC	15 (26.3)	30 (29.1)	37 (22.2)	
Other hormonal contraceptive	13 (22.8)	42 (40.8)	28 (16.8)	
Migraine frequency, n (%)				< 0.001
≤ 1 day/month	–	40 (38.8)	23 (13.8)	
1–4 days/month	–	42 (40.8)	80 (47.9)	
≥ 5 days/month	–	21 (20.4)	64 (38.3)	
Type of migraine, n (%)				< 0.001
Without aura	–	41 (39.8)	103 (61.7)	
With aura	–	62 (60.2)	64 (38.3)	

*Non-MM* non-menstrual migraine, *MM* menstrual migraine, *COC* combined oral contraceptive. A relationship is defined as a serious and intimate monogamous relationship. A regular menstrual cycle is defined as a menstrual cycle duration of 21 to 35 days. Irregular menstrual cycle duration is defined as shorter than 21 days, longer than 35 days or an irregular duration

**Table 2:** Characteristics of the postmenopausal study population

	Postmenopausal control (*n* = 54)	Postmenopausa lmigraine (*n* = 117)	*p*-value
Age, y, mean (SD)	59.9 (6.2)	58.1 (6.7)	0.093
Relationship duration > 1 year, n (%)	54 (100)	115 (98.3)	0.334
BMI, mean (SD)	24.8 (3.6)	24.6 (4.4)	0.731
Migraine frequency, n (%)			
≤ 1 day/month	–	26 (22.2)	
1–4 days/month	–	45 (38.5)	
≥ 5 days/month	–	46 (39.3)	
Type of migraine, n (%)	–		
Without aura	–	68 (58.1)	
With aura		49 (41.9)	

A relationship is defined as a serious and intimate monogamous relationship

Premenopausal controls and MM patients were more likely to have a regular menstrual cycle than to use hormonal contraceptives. Non-MM patients more frequently used combined oral contraceptives or other hormonal contraceptives. Migraine and/or headache was in 38% of MM patients and in 30% of non-MM patients a reason to start using combined oral contraceptives. MM patients mentioned more frequently headache and/or migraine as reason for starting other hormonal contraceptives compared to non-MM patients (respectively 61% and 45%). Furthermore, women with MM were more likely to be irregularly cycling (20%) compared to controls (9%) and non-MM patients (7%).

Women using combined oral contraceptives (COC) were younger than women who were regularly cycling (mean 34.9 and 38.5 years, respectively). The percentage of women with a relationship duration of at least 1 year in the COC group was 89%, which was comparable to the group with a regular menstrual cycle (96%). Furthermore, 82% of participants in the COC group had migraine, compared to 79% of the women with a regular menstrual cycle.

### Primary analysis

**Fig. 2:**
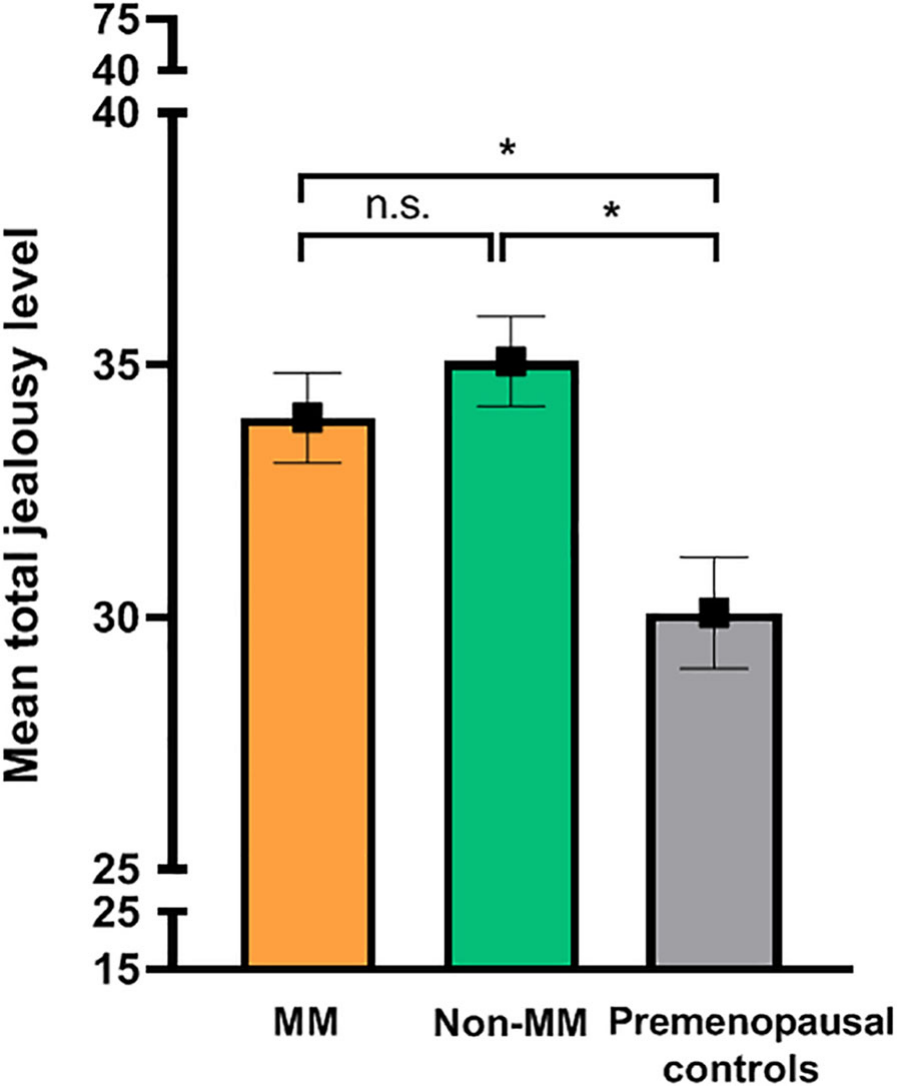
Mean of total jealousy levels controlled for age, relationship duration and hormonal status. Legend: Premenopausal women: MM = menstrual migraine; non-MM = non-menstrual migraine; Premenopausal non-migraine controls. Jealousy levels were determined using the validated Buunks Jealousy scale (score between 15 and 75). Depicted levels are mean ± SEM.* = statistically significant difference, n.s. = non-statistically significant difference

There was a significant difference in mean total self-reported jealousy levels between MM, non-MM and premenopausal control groups, *X*^2^ (2)= 18.05, *p* < 0.001. After adjusting for age, relationship duration and hormonal status, the difference between groups remained statistically significant, *X*^2^ (2)= 18.67, *p* < 0.001. A pairwise comparison with Bonferroni correction revealed that the mean jealousy levels were higher in patients with MM compared to controls (mean difference ± SE: 3.87 ± 1.09, *p* = 0.001), and in non-MM patients compared to controls (4.98 ± 1.18, *p* < 0.001). There was no difference in jealousy levels between the MM and non-MM group (− 1.11 ± 0.93, *p* = 0.705) (see Fig. 2). Age was negatively correlated with jealousy levels, resulting in a decline of 0.49 points per 5 years, *X*^2^ (1) = 5.1, *p* = 0.024. The homogeneity of variance, tested with a Levene’s test of equality of error variances, was violated in this primary analysis (F (2,324) = 8.94, *p* < 0.001). However, using a robust model did not alter the outcome, therefore no adjustments were made to correct for this violation.

### Secondary analyses

**Fig. 3:**
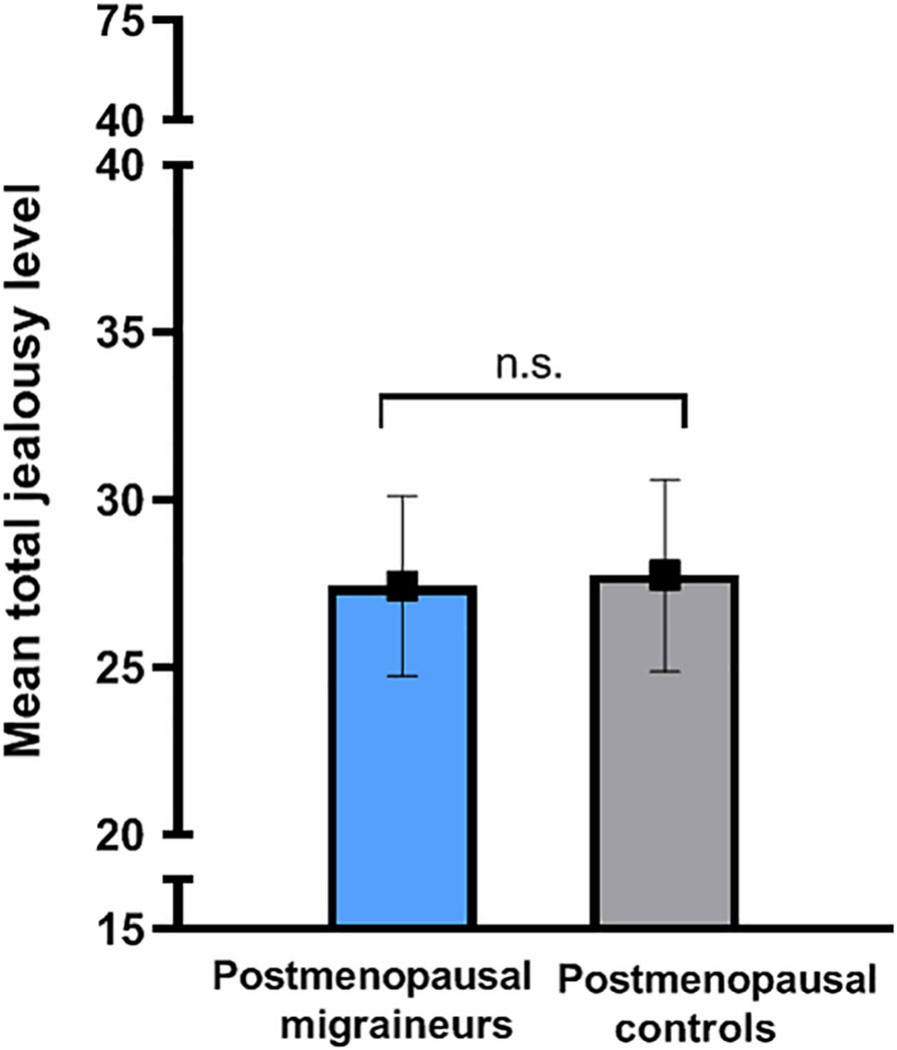
Mean of total jealousy levels controlled for age and relationship duration. Legend: Postmenopausal women: Postmenopausal migraine patients; Postmenopausal non-migraine controls. Jealousy levels were determined using the validated Buunks Jealousy scale (score between 15 and 75). Depicted levels are mean ± SEM. n.s. = non-statistically significant difference

Mean total jealousy levels were similar in postmenopausal migraine patients and controls (mean difference ± SE: -0.41 ± 1.23, p = 0.737). Adjusting for age and relationship duration did not influence the outcome (-0.32 ± 1.24, p = 0.798) (see Fig. 3). Women using COC reported higher jealousy levels compared to women with a regular menstrual cycle (2.32 ± 1.03, p = 0.025). After adding age and relationship duration as covariates, this effect became borderline significant (1.86 ± 1.04, p = 0.073). Women with a relationship duration of at least 1 year scored 4.9 points lower compared to women with a relationship duration of less than 1 year, *X*^2^ (1) = 5.6, p = 0.018. The presence of migraine was associated with an increase of 3.6 points in jealousy levels (*X*^2^ (1) = 8.6, p = 0.003). However, adding migraine status as covariate did not alter the overall effect of using COC on jealousy levels (1.77 ± 1.02, p = 0.081) (Table 3). Migraine attack frequency was not added as covariate as it did not affect jealousy levels in both, the total group of migraine patients (*X*^2^ (5) = 3.14, p = 0.678) and the subgroup of premenopausal migraine patients (*X*^2^ (5) = 5.53, p = 0.355).

**Table 3:** Comparison of jealousy levels between women using COCs and women with a regular menstrual cycle

	Combined oral contraceptive (*n* = 82)	Regular menstrual cycle (*n* = 116)	*p*-value	
Unadjusted (mean ± SE)	34.3 ± 0.79	31.9 ± 0.67	0.025	
Adjusted for age and relationship duration (mean ± SE)	36.1 ± 1.16	34.2 ± 1.13	0.073	
Adjusted for age, relationship duration and migraine status (mean ± SE)	35.1 ± 1.18	33.4 ± 1.14	0.081	

### Exploratory analyses

Mean differences in the three sub-type jealousy levels (reactive jealousy, preventive jealousy and anxious jealousy) were analyzed for the premenopausal groups as exploratory analyses. A pairwise comparison with Bonferroni correction revealed that both MM and non-MM groups reported higher levels compared to the premenopausal control group for the reactive jealousy sub-type (mean difference ± SE: 1.89 ± 0.64, *p* = 0.010 and 1.97 ± 0.70, *p* = 0.014, respectively). Similarly, both MM and non-MM groups reported higher anxious jealousy levels compared to premenopausal controls (1.24 ± 0.49, *p* = 0.035 and 1.83 ± 0.53, *p* = 0.002, respectively). No statically significant difference was found in mean preventive jealousy levels between premenopausal controls and MM patients (− 0.74 ± 0.35, *p* = 0.104). Non-MM patients reported higher mean preventive jealousy scores compared to premenopausal controls (1.18 ± 0.38, *p* = 0.005).

## Discussion

Premenopausal women with migraine in a relationship have significantly higher jealousy scores than controls in this study. This is independent from whether they experience menstrually-related attacks and the effect disappears after menopause. Our hypothesis is that this association between migraine and increased jealousy is due to the effect of estrogen. Previous research showed estrogen levels to be higher in women with MM compared to controls during most phases of the menstrual cycle, and with only small differences between MM and non-MM patients [7, 8]. As estrogen levels will be low in the postmenopausal stage of life we expected that difference in jealousy levels would diminish and indeed we did not find differences when comparing postmenopausal female migraine patients with controls supporting our hypothesis. Interestingly, we did not find a significant difference in jealously between patients with menstrually-related attacks (MM) and those without menstrually-related attacks (non-MM). We imagine that there might be one important explanation for this, namely the inaccuracy of non-diary self-reported MM or non-MM diagnosis. In a recent study, we asked 104 female migraine patients whether their attacks were associated with the menstruation and then collected prospective e-diaries. In this study, we showed women’s self-reported diagnoses had a positive predictive value of 65% and negative predictive value of 50%. Sensitivity was 80% and specificity 33% [22]. Accurate MM diagnoses are difficult to obtain even when prospective diaries are collected. Previous research has shown that current ICHD-3 diagnostic criteria for MM reached maximum sensitivity only for three menstrual cycles, although specificity increased with more cycles of data collection [23]. Thus, accuracy of self-reported menstrual-related migraine diagnosis is poor in female migraine patients and we suggest to reconsider the ICHD-3 criteria for menstrual migraine where no prospective diary data is required anymore to confirm MM.

Are there alternative explanations for our findings? The effect of a disabling chronic disease on the quality of life might explain the higher jealousy response within romantic relationships in female migraine patients. The most recent Global Burden of Disease study ranked migraine as the second most disabling disease worldwide [24]. Previous studies showed that migraine patients scored lower on health-related quality of life domains than controls, such as social functioning and mental health [25, 26]. Female migraine patients might have less social interaction compared to their partners, both during a migraine attack due to severe headache and disabling associated symptoms, but also outside migraine attacks due to an adjusted lifestyle trying to prevent migraine attacks. One could imagine that a disbalance in social interactions in a romantic relationship may induce jealousy towards a partner. Studying the association between other disabling chronic diseases and jealousy within romantic relationships may be of interest in this light. Although postmenopausal women with migraine are limited in social activities, their jealousy response is comparable to that of postmenopausal controls, suggesting that impaired social functioning only partially contributes to the difference in jealousy between younger migraine patients and controls. Several population-based studies have analysed the prevalence of disabling pain disorders and associated risk factors. Separated or divorced status is consistently associated with an increased risk of (chronic) pain in women [27–29]. Additionally, separation and divorce have been suggested to be a risk factor for worsening outcomes in pain disorders with persisting pain [30]. This knowledge might be helpful in understanding the potential adverse effects of migraine on the relationships of patients, such as jealousy and potentially divorce.

A recent meta-analysis on personality of migraine patients has shown higher risk for neuroticism and harm avoidance, and for low self-directedness and extraversion in migraineurs [31], which hypothetically may be involved in more pronounced reactive and anxious jealousy scores than preventive scores for MM patients.

Women using combined oral contraceptives reported higher jealousy compared to non-using women with a regular menstrual cycle, which is congruent with previous studies [13, 20]. With this, our study contributes to the existing literature by using a different study population and adjusting for relevant covariates, which limits potential confounders and increases the validity. Participants in our study were older and had a longer relationship duration compared to participants in other studies, who were students with a mean age of 22 years and a mean relationship duration of 1 year [13, 20]. The higher jealousy levels in women using combined oral contraceptives might be caused by an effect of estrogen, which is suggested to influence jealous behavior [14, 15]. Progesterone dose in combined oral contraceptives is shown to be unrelated to reported jealousy, but combined oral contraceptives with higher doses of ethinyl estradiol are associated with higher jealousy compared with formulations with lower ethinyl estradiol doses [14, 15].

This study has a number of strengths. A large number of participants from the reliable LUMINA cohort were recruited. Furthermore, a validated jealousy scale was used, with a Cronbach’s alpha of 0.843 indicating a very good internal consistency. However, some limitations of our study should be mentioned. Firstly, a considerable part of invited women were classified as non-responders. The non-response rate could partially be explained by women who were not eligible for participation, e.g. as they had no romantic partnership at the time of the study but refrained from informing the investigators. Secondly, as indicated the MM and non-MM diagnoses in this study were not based on diary data as this is not a requirement anymore in the ICHD-3 classification. In addition, the phase of the menstrual cycle at the time of completing the questionnaire is unknown. In a prior study, higher jealousy levels were found in the fertile phase compared to the non-fertile phase of the menstrual cycle. However, this effect became marginally significant when comparing the menstrual cycle phases in partnered women [13]. Since we included only partnered women, this is thus thought to be of less importance for our results. Additionally, since the percentage of women with a regular menstrual cycle in the MM and control group is comparable (41% vs. 42% respectively), the amount of women in the fertile and non-fertile phase is expected to be equally distributed in these groups.

## Conclusions

Our study is the first to show that young migraine women are more jealous within a romantic partnership than non-migraine women. We suggest estrogen to play an important role in this relationship. Future research is needed on establishing the role of estrogen in women with migraine as this may provide important treatment options for this incapacitating disorder. We encourage physicians treating patients with migraine to pay attention to aspects of social functioning.

## Supplementary information

**Additional file 1.**

## Data Availability

Data not published within the article is available from the corresponding author on reasonable request.

## Abbreviations

COC: Combined oral contraceptives
LUMINA: Leiden University Medical Center MIgraine Neuro Analysis
MM: Menstrual migraine
MRM: Menstrually-related migraine
non-MM: Non-menstrual migraine
PMM: Pure menstrual migraine
