# Supplementary material for: Jealousy in women with migraine: a cross-sectional case-control study
Source: J Headache Pain. 2020 May 11;21(1):51. doi: 10.1186/s10194-020-01114-5 (PMC7216716; doi:10.1186/s10194-020-01114-5)
Supplement: Supplementary file 1 — Additional file 1. [file 10194_2020_1114_MOESM1_ESM.docx]

**ADDITIONAL INFORMATION**

**LUMINA Background information**

Dutch migraine patients aged 18-80 years were recruited via nationwide public announcement, advertising in lay press and our research website ([www.lumc.nl/hoofdpijn](file:///\\vf-d3-home\d3home$\gmterwindt\GISELA\2019\Katie%20Linstra\www.lumc.nl\hoofdpijn)). They were considered eligible after a two-step inclusion process using validated questionnaires via the dedicated Leiden University MIgraine Neuro-Analysis (LUMINA) website. Additionally, patients attending our outpatient headache clinic were invited to participate by a letter. Patients were first asked to fill out a validated web-based screening questionnaire with a sensitivity of 0.93 and specificity of 0.36.^1^ Patients who fulfilled the screening criteria, were sent a validated web-based extended migraine questionnaire^2^, based on the International Classification of Headache Disorders criteria (previously ICHD-2, now ICHD-3 version) criteria.^3^ The specificity of the second questionnaire was 0.95 and sensitivity was 0.45.^2^ This questionnaire is accessible for patients via our research website and is described in English in detail by van Oosterhout et al. 2011.^2^ We consider the cohort a well-defined web-based cohort. Four percent of subjects were included from our headache outpatient clinic and 87% of the participants were previously diagnosed with migraine by a physician. In addition to questions that were necessary to diagnose migraine accurately, the extended questionnaire also included items on demographic factors, aura and headache characteristics, acute and prophylactic headache medication use, and allodynia. Participants unable to use the web-based questionnaires due to lack of the needed internet skills were allowed to fill out the questionnaires on paper.

**References**

1. Launer LJ, Terwindt GM, Ferrari MD. The prevalence and characteristics of migraine in a population-based cohort: the GEM study. Neurology. 1999;53(3):537-42.

2. van Oosterhout WP, Weller CM, Stam AH, et al. Validation of the web-based LUMINA questionnaire for recruiting large cohorts of migraineurs. Cephalalgia. 2011;31(13):1359-67.

3. Headache Classification Committee of the International Headache Society (IHS). The International Classification of Headache Disorders, 3rd edition. Cephalalgia. 2018;38(1):1-211.
